# Gut microbiota dysbiosis, sarcopenia, osteoporosis and osteosarcopenia in older people: A systematic review protocol

**DOI:** 10.1371/journal.pone.0313193

**Published:** 2025-01-02

**Authors:** Nailton José Neto, Mário Duarte Brito, Cristiano dos Santos Gomes, Luana Caroline de Assunção Cortez Corrêa, Gerlane Coelho Bernardo Guerra, Ricardo Oliveira Guerra

**Affiliations:** 1 Graduate Program in Health Sciences, Federal University of Rio Grande do Norte, Natal, Brazil; 2 Physical Therapy Department, University of Pernambuco, Petrolina, Brazil; 3 Graduate Program in Physical Therapy, Physical Therapy Department, Federal University of Rio Grande do Norte, Natal, Brazil; 4 Department of Biophysics and Pharmacology, Federal University of Rio Grande do Norte, Natal, Brazil; McMaster University, CANADA

## Abstract

**Introduction:**

Sarcopenia and Osteoporosis are two prevalent conditions in the older population and are defined by low strength, muscle quality/volume and low Bone Mineral Density, respectively. When there is a concomitant presence of both, there is a novel musculoskeletal condition called Osteosarcopenia. These conditions adversely affect quality of life and elevate the risk of fractures, disability, and mortality among older individuals. Dysbiosis of the gut microbiota is the impairment of the mutualistic relationship between microorganisms, metabolic products and the host’s immune system. Gut microbiota dysbiosis could be intricately linked to sarcopenia and osteoporosis, shedding light on the complex microbiota-gut-bone-muscle axis. Furthermore, the intestinal microbiota experiences a notable decline in beneficial microorganisms as part of the aging process. The relationship between dysbiosis of the intestinal microbiota in older people and sarcopenia, osteoporosis or osteosarcopenia is still unclear. This review protocol aims to systematically review the literature and compile evidence on the influence of gut microbiota dysbiosis on musculoskeletal function in older people with sarcopenia, osteoporosis or osteosarcopenia.

**Methods/Analysis:**

This systematic review will analyze observational studies that have investigated the relationship between the effects of gut microbiota dysbiosis and sarcopenia, osteoporosis and osteosarcopenia in older people aged 65 and over. Studies will be retrieved from PubMed/MEDLINE, EMBASE, Scopus, Web of Science and the Cochrane Library. Outcome measures will include body composition for diagnosing osteoporosis and screening for sarcopenia/osteosarcopenia by any criteria. Data synthesis will involve quantitative analysis using summary measures. If sufficient studies, homogeneity and heterogeneity analysis will be performed to conduct Meta-analysis and pooled OR, RR and HR measures will be provided.

## Introduction

The demographic shift towards an older population worldwide has highlighted the need to address age-related health conditions, particularly those affecting musculoskeletal and metabolic health [1]. Among these, sarcopenia, osteoporosis, and the more recently recognized condition of osteosarcopenia (OS) (the simultaneous occurrence of sarcopenia and osteoporosis/osteopenia) represent significant health burdens [2]. These conditions not only impair quality of life, but also increase susceptibility to fractures, disabilities and mortality in older adults [3]. Through mechanical connections at the macroscopic and microscopic levels (e.g. secretion of osteokines and myokines), bone and muscle tissues share common changes in ageing and therefore osteosarcopenia increases adverse outcomes [2]. In the older population, the prevalence of sarcopenia and osteoporosis ranges from 10% to 27% and 21.7%, respectively [4, 5]. Both are the most significant musculoskeletal conditions in human ageing. The prevalence of osteosarcopenia was established at 1.5–65.7%, and 1) females, 2) advanced age and 3) fracture were identified as risk factors for OS [6].

Sarcopenia is a common age-related disorder involving the loss of muscle mass and a decline in strength and function [7]. For the European Working Group on Sarcopenia in Older People (EWGSOP), sarcopenia is probable when there is low muscle strength; it is confirmed when there is low muscle quantity or quality; and it is severe when there is low physical performance [8]. Diagnostic measures for sarcopenia are based on muscle strength assessment, imaging tests (e.g. dual energy X-Ray absorptiometry) and physical performance (e.g. short physical performance battery or handgrip strength) [9–11]. Osteoporosis is diagnosed by T-scores ≤ -2.5 standard deviation (SD) below the average Bone Mineral Density (BMD) of the reference population of the same sex [6, 12].

The human Gut Microbiota (GM), comprising 10 to 100 trillion microorganisms, plays essential roles in intestinal, immunological, and nutritional functions, as well as in overall human body homeostasis [13]. Aging process primarily affects the IM through the loss of beneficial microorganisms, influenced by factors like diet, medication, sedentary lifestyles, and chronic diseases. According to Gagliardi et al, dysbiosis of the intestinal microbiota is characterized by the lack of the mutualistic relationship between microorganisms, metabolic products and the host’s immune system [14].

This dysbiosis or dysfunction of the gut microbiome related to aging may contribute to various pathologies, including intestinal, musculoskeletal and metabolic issues [15]. Research points to gut microbiota dysbiosis, a disturbance in microbial communities within the gut, as a potential key player in the pathogenesis of these age-related conditions [16, 17]. These changes can contribute to an inflammation-related state often seen in aging, known as "*inflammaging*," potentially impacting the development and progression of musculoskeletal disorders such as sarcopenia, osteoporosis and osteosarcopenia [18]. Gut microbiota dysbiosis could be intricately linked to sarcopenia and osteoporosis, shedding light on the complex microbiota-gut-bone-muscle axis [19–22]. Dysbiosis could affect this axis through various mechanisms, such as altered metabolic functions, impaired synthesis of short-chain fatty acids (SCFAs), and disrupted hormone regulation, crucial for bone and muscle health [23]. Disruptions in these pathways due to dysbiosis might lead to the simultaneous degradation of muscle and bone, characteristic of osteosarcopenia.

Although recent studies have investigated the relationship between gut microbiota dysbiosis and musculoskeletal function [17, 24], there are still no studies that have aimed to explore the effect of gut microbiota dysbiosis on sarcopenia, osteoporosis and osteosarcopenia together. By reviewing the evidence systematically, this study intends to highlight potential pathways through which the gut microbiota influences these conditions and to identify gaps in the current knowledge that warrant further investigation. The findings of this review are expected to pave the way for novel interventional strategies that could mitigate the impacts of these debilitating conditions in the aging population. Thus, the aim of our study is to systematically review the literature on the influence of gut microbiota dysbiosis on musculoskeletal function in older people with sarcopenia, osteoporosis, or osteosarcopenia.

## Material and methods

### Study design

This review is registered in the International Prospective Register of Systematic Reviews (PROSPERO) under the name “Gut microbiota dysbiosis, Sarcopenia, Osteoporosis and Osteosarcopenia in Older People: A Systematic Review Protocol” (CRD42023455040). It will also be conducted following the protocols of the Preferred Reporting Items for Systematic Reviews and Meta-Analyses (PRISMA-P) [25].

### Eligibility criteria

This review will include observational studies (case-control, cross-sectional or cohort) that set out to assess the relationship between dysbiosis of the gut microbiota and the presence of sarcopenia, osteoporosis or osteosarcopenia in older people aged 65 and over. Studies must provide diagnostic information for sarcopenia by any criterion (e.g. European Working Group on Sarcopenia in Older People or The Asian Working Group for Sarcopenia). For osteoporosis, studies must provide screening measures based on Standard Deviation T-Scores; for osteosarcopenia, studies must meet the criteria for categorization of their subjects based on the concomitant presence of sarcopenia and osteoporosis.

### Outcomes

The main outcome measures should be acquired by assessing body composition through suitable imaging diagnostics (e.g. Dual Energy X-ray Absorptiometry—DXA; Magnetic Resonance Imaging—MRI). For sarcopenia screening, studies should be grounded on measures such as the Strength, Assistance with walking, Rising from a Chair, Climbing Stairs, and Falls Questionnaire (SARC-F). The severity of Sarcopenia should be measured by Physical Performance measures (e.g. Short Physical Performance Battery—SPPB; Handgrip Strength—HGS). Such measures are unbiased and independent of the study results. In addition, any included studies should provide diagnostic information for these conditions, thus eliminating floor or ceiling effects by relying on established diagnostic criteria.

Secondary outcomes will include alpha and beta diversity of the intestinal microbiota, taxonomic groups (kingdom, phylum, class, order, family, genus, and species), and changes in inflammatory markers associated with intestinal microbiota and musculoskeletal function, including Interleukin 6 and 10, C-Reactive Protein, and Tumor Necrosis Factor Alpha.

### Comparisons

If information is available from the selected studies, we will compare the influence of gut microbiota dysbiosis on musculoskeletal function in older people without sarcopenia, osteoporosis or osteosarcopenia.

### Search strategy

Searches will be conducted on the electronic databases PubMed/MEDLINE, EMBASE, Scopus, Web of Science, and the Cochrane Library. No search limiters will be applied for language or date. A preliminary search will be conducted in PubMed to map relevant keywords related to the proposed topic and further refine the search strategy. The identification of eligible studies will be conducted in three stages: initial search, title and abstract screening, and full-text manuscript review. The search strategy will be developed under the guidance of a professional systematic review librarian and will involve a combination of relevant keywords, Medical Subject Headings (MeSH terms), and Boolean operators to include all relevant studies pertaining to elderly and human studies. The first step applied to the selected articles in the search strategy will be the removal of duplicate references using the reference management software Rayyan [26].

The preliminary search results for the MeSH terms listed in the PubMED/MEDLINE electronic database are shown in **Table 1**.

**Table 1:** PECO strategy, MeSH codes and number of studies indexed in the PUBMED/MEDLINE electronic database (Search carried out in May 17, 2024).

PECO Strategy	MeSH code	No. of Studies
**Population**	aged OR elderly OR elderly people OR older adults OR older people	6.239.124
**Exposure**	gastrointestinal microbiota dysbiosis OR gut microbiota dysbiosis	14.547
**Condition**	osteosarcopenia OR sarcopenia OR osteoporosis OR osteopenia OR bone diseases, metabolic OR dynapenia OR dynapaenia	160.029
**Outcomes**	Body Composition OR Sarcopenia OR Bone Mineral Density OR Dual-energy X-ray Absorptiometry (DXA) OR Physical performance	366.158

**Note:** AND/OR: Boolean operators; MeSH: Medical Subject Headings

The preliminary search for studies available in the PubMED/MEDLINE electronic database is shown below in **Table 2**.

**Table 2:** Search results based on a search algorithm for studies indexed in the PUBMED/MEDLINE electronic database.

Search Date	Search Algorithm	No. of studies
**May 17, 2024**	(aged OR elderly OR elderly people OR older adults OR older people) AND (osteosarcopenia OR sarcopenia OR osteoporosis OR osteopenia OR bone diseases, metabolic OR dynapenia OR dynapenia) AND (gastrointestinal microbiota dysbiosis OR gut microbiota dysbiosis) AND (Body Composition OR Sarcopenia OR Bone Mineral Density OR DXA OR physical performance)	**25**

### Study selection

The review, screening, eligibility, and inclusion phases in the meta-analysis will be conducted blindly and independently by two junior authors (MDB and NJN) and adjudicated by the third senior author (ROG). Any conflicts regarding the studies will be resolved through discussion among the authors before data inclusion in the final analysis. The files devoid of duplicate articles will be uploaded and processed for screening through the web application Rayyan. Initially, the titles and abstracts of the studies will be assessed for relevance and selected for eligibility. Eligible studies will be evaluated in the second phase for inclusion and quality criteria, and in the final phase, eligible manuscripts will be reviewed and read in full for subsequent data extraction. This process will be reported in the final systematic review using the Preferred Reporting Items for Systematic Reviews and Meta-Analyses (PRISMA) flow diagram [27]. The PRISMA protocol checklist pertaining to this protocol is available in **S1 Table**.

### Data extraction

Quantitative data will be extracted from studies included in the full-text review by a professional biostatistician. Data extracted for meta-analysis will include those relevant to the specified primary and secondary outcomes in the systematic review. This will encompass patient demographics, interventions, comparators, and outcome measures such as body composition, physical performance, diversity of intestinal microbiota, and changes in inflammatory markers. Extracted data will be independently verified by the senior author, and any discrepancies arising will be resolved through discussion with the biostatistician. A data extraction tool developed by the reviewers (e.g., **S2 Table**) will be utilized. The data extraction tool may be modified and revised as necessary during the data extraction process for each included source of evidence. Any modifications will be detailed in the final systematic review. If needed, authors of articles will be contacted to request missing or additional data.

### Quality assessment

To reduce the risk of bias, each article will undergo initial title and abstract screening in parallel by two independent and blinded reviewers. The opinion of the senior author will be sought as needed. The Newcastle Ottawa Scale (NOS) tool will be used to assess bias risk in observational studies included in the review.

To address meta-bias, in addition to the strategies mentioned earlier, a sensitivity analysis will be conducted to assess the potential impact of studies with high risk of bias on the overall results of the meta-analysis. This will involve the sequential exclusion of studies with high risk of bias and comparison of results before and after exclusion to determine if they significantly influence the conclusions of the review.

#### Analysis

*Data synthesis and meta-analysis*. The study screening summary will be presented using the PRISMA flowchart. The findings from the systematic review will be organized in a clear and transparent manner in line with the PRISMA 2020 statements established for systematic reviews and meta-analyses. The data from the studies will be synthesized quantitatively, respecting the criteria: relevance to the outcomes of interest, availability of numerical or categorized data, homogeneity of results and interventions, and absence of significant bias.

The evaluation of outcome measures, body composition, physical performance, diversity of intestinal microbiota, and inflammatory markers will be presented through summary measures such as mean and standard deviation. To ensure comparability and facilitate the analysis and interpretation of results among studies, data may be standardized on a common scale. Additionally, evaluated interventions may be grouped based on specific categories such as taxonomic groups or type of intervention (probiotics, prebiotics, diet).

If possible, a meta-analysis will be conducted with the included studies. Chi-squared test (X^2^) will be used for homogeneity between studies (p-value <0.1) [28] and the I^2^ test (l^2^ ≤50%) for heterogeneity [29]. Pooled OR, RR or HR and 95% Confidence Intervals (CI) will be calculated. The fixed-effects model will be used to conduct the meta-analysis using Review Manager (RevMan) software. Data will be statistically significant when p-value ≤ 0.05.

If quantitative synthesis is not appropriate due to substantial heterogeneity among studies or lack of adequate numerical data, a narrative synthesis of results will be conducted. In this case, studies will be grouped in tables and graphs, and qualitative results will be examined to identify common trends, such as variations in intestinal microbiota composition associated with different states of sarcopenia, osteoporosis, and osteosarcopenia, along with shared risk factors among these conditions.

#### Certainty of cumulative evidence

To assess the certainty of cumulative evidence related to intestinal microbiota dysbiosis, sarcopenia, osteoporosis, and osteosarcopenia in older individuals, we will employ the GRADE (Grading of Recommendations, Assessment, Development, and Evaluation) system [30]. This systematic approach will allow for robust analysis, considering factors such as consistency of results, precision of estimates, potential biases, and applicability of findings. By using GRADE, we will be able to quantify the level of confidence in the evidence, providing a basis for inferences and recommendations.

## Discussion

The advancement of the human aging process raises issues of great relevance that impact musculoskeletal function. The hypothesis that gut microbiota dysbiosis influences sarcopenia, osteoporosis, and osteosarcopenia in older adults is supported by emerging evidence linking age-related changes in the gut microbiome with musculoskeletal function [15]. However, the current body of literature is still evolving, and while our systematic review aims to aggregate existing data. Studies have demonstrated that dysbiosis may contribute to conditions such as “inflammaging,” a low-grade, chronic inflammatory state associated with aging, which could exacerbate both muscle loss and bone degradation [18]. Therefore, the hypothesized link between dysbiosis and sarcopenia/osteoporosis appears biologically plausible. The relationship between the gut microbiota and the expected outcomes remains an area of active research. The findings of this review are expected to contribute to a more holistic understanding of how microbiota modulations can affect musculoskeletal health in older adults.

Osteosarcopenia, a condition characterized by the simultaneous presence of sarcopenia and osteoporosis, implies restrictions in functionality, increased risk of falls and fractures, as well as representing a burden on healthcare systems. This systematic review aims to elucidate the underlying mechanisms of the microbiota-gut-bone-muscle axis, identifying potential pathways through which the influence of intestinal microbiota affects sarcopenia, osteoporosis, and osteosarcopenia. It is expected that the systematic analysis of the literature will provide robust scientific evidence capable of guiding the development of new intervention strategies and actions to mitigate the impacts of these conditions. The potential heterogeneity and variable methodological quality of the studies to be included may pose challenges to this systematic review and eventual meta-analysis.

## Strengths and limitations of this study

- This systematic review and meta-analysis will investigate the impact of intestinal microbiota dysbiosis on musculoskeletal function in older people.
- Clarifying the convergent pathways of sarcopenia, osteoporosis, and osteosarcopenia with intestinal microbiota dysbiosis offers novel therapeutic perspectives.
- The limited number of studies linking osteosarcopenia with intestinal microbiota dysbiosis may constrain the overall conclusions of the review.
- Heterogeneity among the included studies may preclude the possibility of conducting meta-analyses.

## Supporting information

S1 Table PRISMA-P (Preferred Reporting Items for Systematic review and Meta-Analysis Protocols) 2015 checklist: Recommended items to address in a systematic review protocol. (DOC)

S2 Table Data extraction tool. (DOCX)
