# Supplementary material for: Gut microbiota dysbiosis, sarcopenia, osteoporosis and osteosarcopenia in older people: A systematic review protocol
Source: PLoS One. 2025 Jan 2;20(1):e0313193. doi: 10.1371/journal.pone.0313193 (PMC11695030; doi:10.1371/journal.pone.0313193)
Supplement: S2 Table — (DOCX) [file pone.0313193.s002.docx]

**Supplement S3:** Data Extraction Tool

| **1. General Information** | **Extracted Data** |
| --- | --- |
| Study Title |  |
| Study Author(s) |  |
| Year of Publication |  |
| Publication Source (journal, conference, etc.) |  |
| Country/Region of Study Origin |  |
| **2. Study Characteristics** |  |
| Study Design (e.g. cross-sectional study, longitudinal study, cohort study) |  |
| Sample Size (n) |  |
| Study Duration |  |
| **3. Studied Population** |  |
| Total Number of Participants |  |
| Age Range of Participants |  |
| Gender |  |
| Ethnicity |  |
| **4. Interventions** |  |
| Prebiotics, Probiotics, Synbiotics (composition, mode of administration, and form of consumption) |  |
| Prebiotic: Dose/Duration |  |
| **5. Outcome Measures for Sarcopenia, Osteoporosis, Osteosarcopenia** |  |
| Diagnostic Criteria for Sarcopenia, Osteoporosis, Osteosarcopenia (EWGSOP, AWGS, dual-energy X-ray absorptiometry (DXA), T-scores, bioelectrical impedance analysis) |  |
| Primary Outcomes (body composition; physical performance) |  |
| Secondary Outcomes (inflammatory markers) |  |
| Observed Results (or absence of results) |  |
| **6. Outcome Measures for Intestinal Microbiota** |  |
| Methods for Identifying Intestinal Microbiota (e.g. 16S rRNA sequencing) |  |
| Composition of Intestinal Microbiota |  |
| Diversity of the Intestinal Microbiota |  |
| Short-Chain Fatty Acids |  |
| Observed Results (or absence of results) |  |
| **7. Study Conclusions** |  |
| Main Conclusions and Findings Highlighted by the Authors |  |
| Study Limitations Identified by the Authors |  |
